# Supplementary material for: ATP-dependent reorientation on a nucleosome primes INO80 for DNA translocation
Source: bioRxiv. 2026 Aug 14:2026.08.11.744281. Preprint. [Version 1] doi: 10.64898/2026.08.11.744281 (PMC13640691; doi:10.64898/2026.08.11.744281)
Supplement: Supplementary file 1 [file NIHPP2026.08.11.744281v1-supplement-3.pdf]

bioRxiv preprint doi: https://doi.org/10.64898/2026.08.11.744281; this version posted August 14, 2026. The copyright holder for this preprint (which was not certified by peer review) is the author/funder, who has granted bioRxiv a license to display the preprint in perpetuity. It is made available under a CC-BY 4.0 International license.

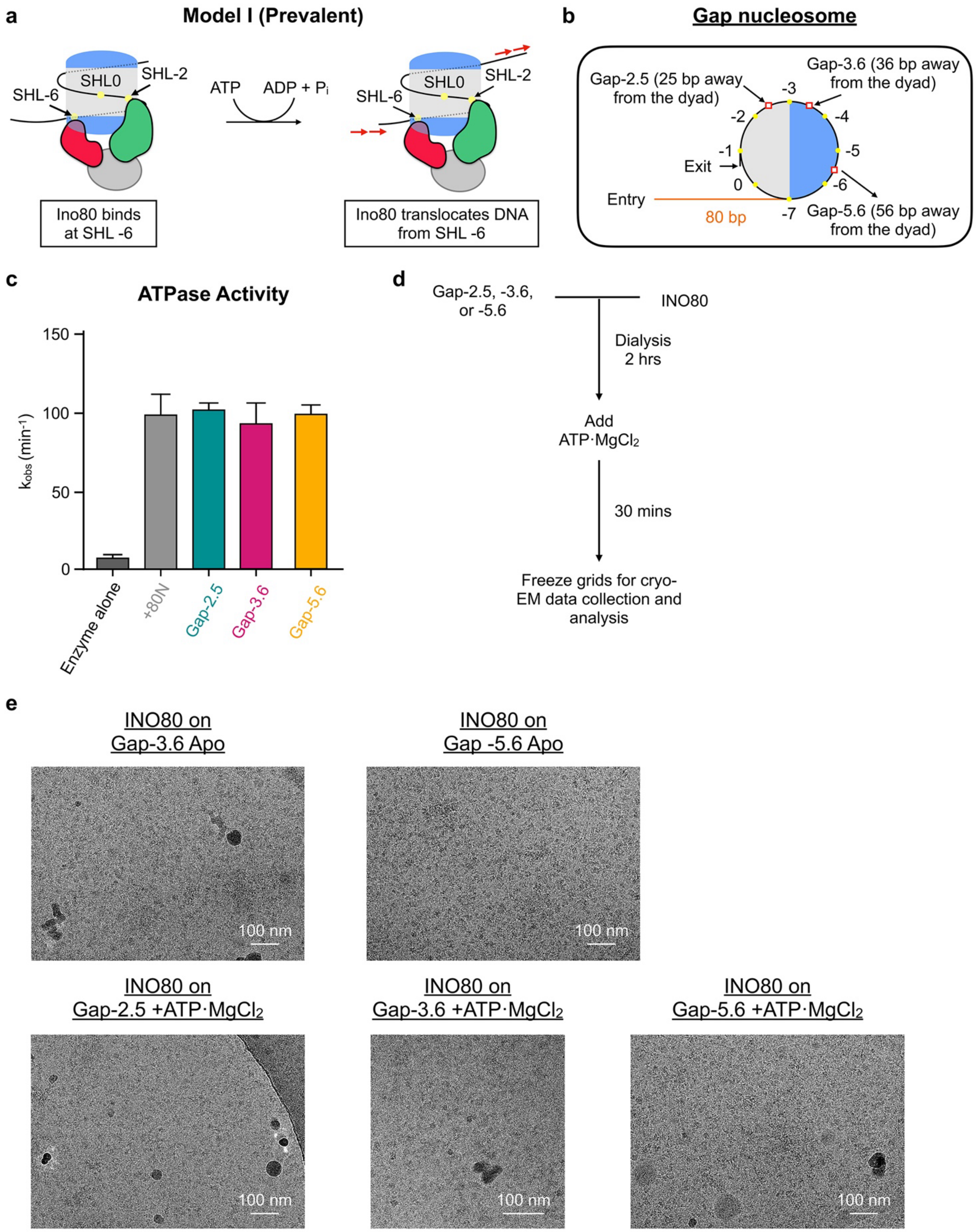

Supplementary Figure 1 Preparation of INO80/Gap nucleosome samples.
a) Previous model of INO80-mediated nucleosome remodeling. INO80 binds nucleosomes at SHL -6 and directly translocates DNA from this position. **b)** Schematic of a +80 nucleosome containing DNA gaps at SHL -2.5, -3.6, or -5.6. Gap positions are labeled by red boxes. **c)** ATPase activity of INO80 on WT nucleosome, Gap-2.5, Gap-3.6, and Gap -5.6 nucleosomes. **d)** Workflow for preparation of cryo-EM samples. **e,** Representative cryo-EM micrographs of the INO80-Gap nucleosome samples.

bioRxiv preprint doi: https://doi.org/10.64898/2026.08.11.744281; this version posted August 14, 2026. The copyright holder for this preprint (which was not certified by peer review) is the author/funder, who has granted bioRxiv a license to display the preprint in perpetuity. It is made available under a CC-BY 4.0 International license.

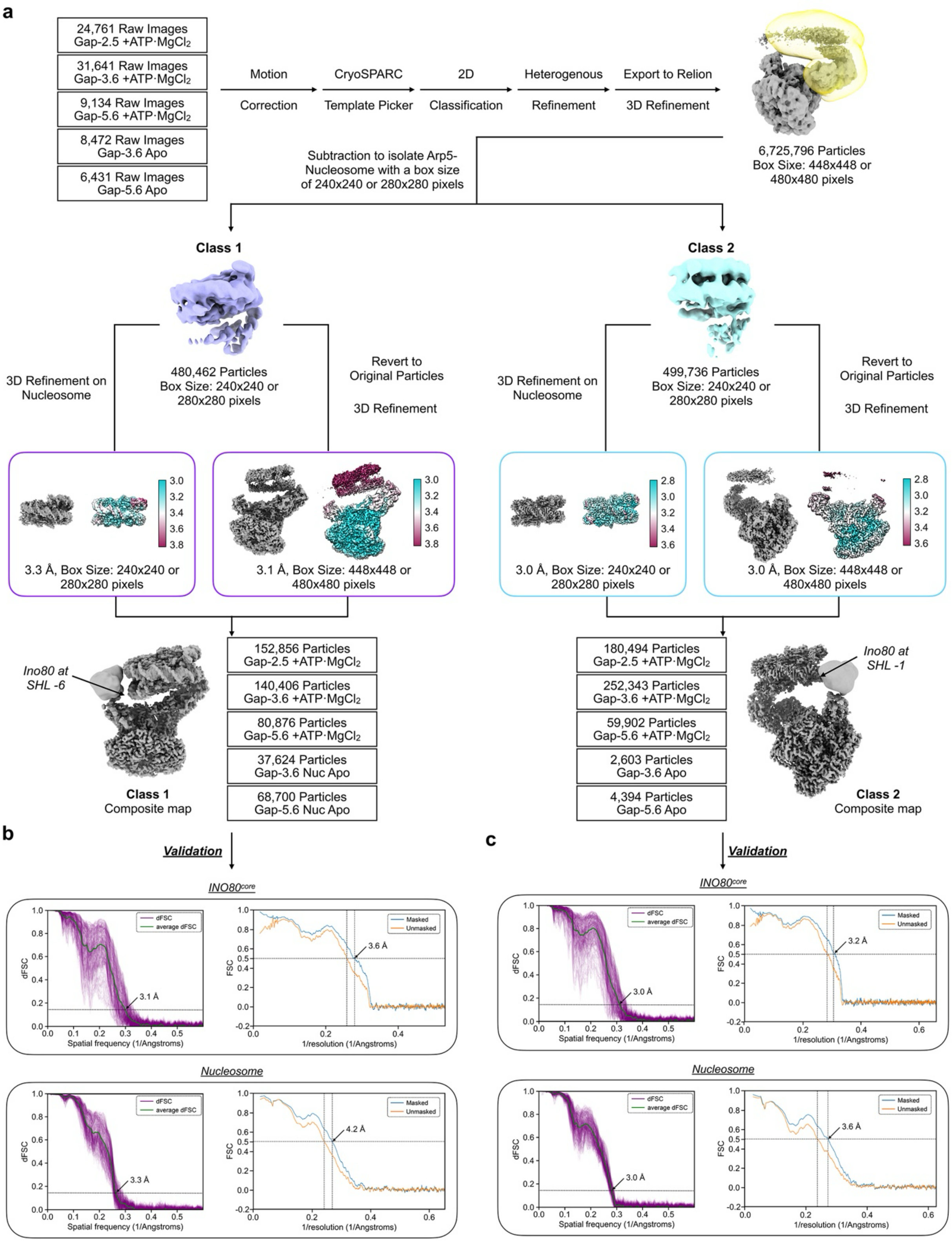

Supplementary Figure 2 Cryo-EM image processing of the INO80/gapped nucleosome datasets. **a)** Image processing workflow for the combined INO80/gap nucleosome cryo-EM datasets. Composite maps are generated following the same procedure. **b)** Resolution estimation from directional Fourier shell correlation (dFSC, 0.143 criteron, left) and model-to-map FSC curves (FSC = 0.5 criterion) of INO80 core (upper) and nucleosome (bottom) of Class 1 composite map. **c)** Resolution estimation from dFSC (left) and model-to-map FSC (right) of INO80 core (upper) and nucleosome (bottom) of Class 2 composite map. Estimated resolutions are marked.

bioRxiv preprint doi: https://doi.org/10.64898/2026.08.11.744281; this version posted August 14, 2026. The copyright holder for this preprint (which was not certified by peer review) is the author/funder, who has granted bioRxiv a license to display the preprint in perpetuity. It is made available under a CC-BY 4.0 International license.

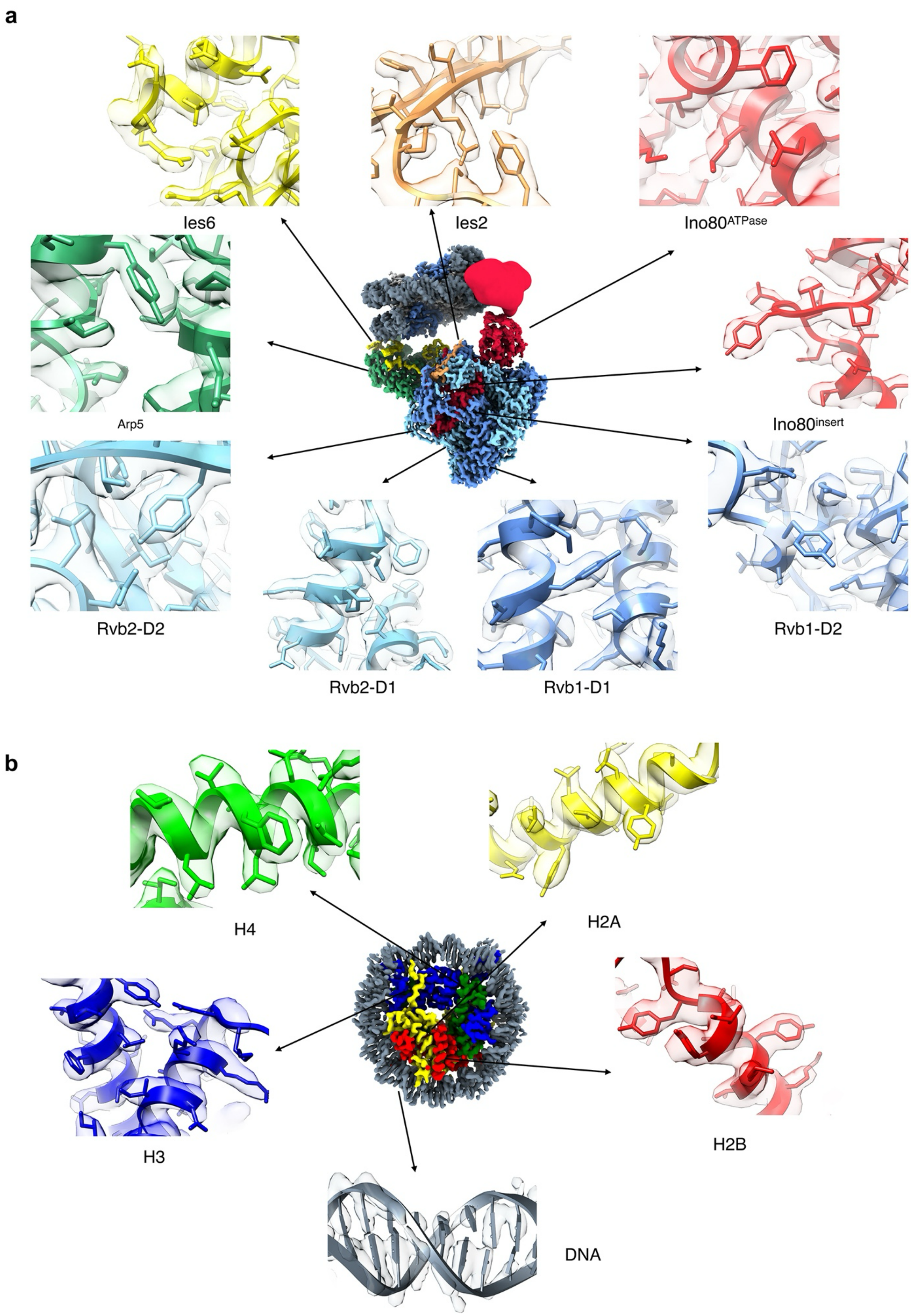

Supplementary Figure 3 Representative densities of the INO80-Gap nucleosome complex. Representative densities of Class 2 composite map. **a**) INO80 core and **b)** nucleosome.

bioRxiv preprint doi: https://doi.org/10.64898/2026.08.11.744281; this version posted August 14, 2026. The copyright holder for this preprint (which was not certified by peer review) is the author/funder, who has granted bioRxiv a license to display the preprint in perpetuity. It is made available under a CC-BY 4.0 International license.

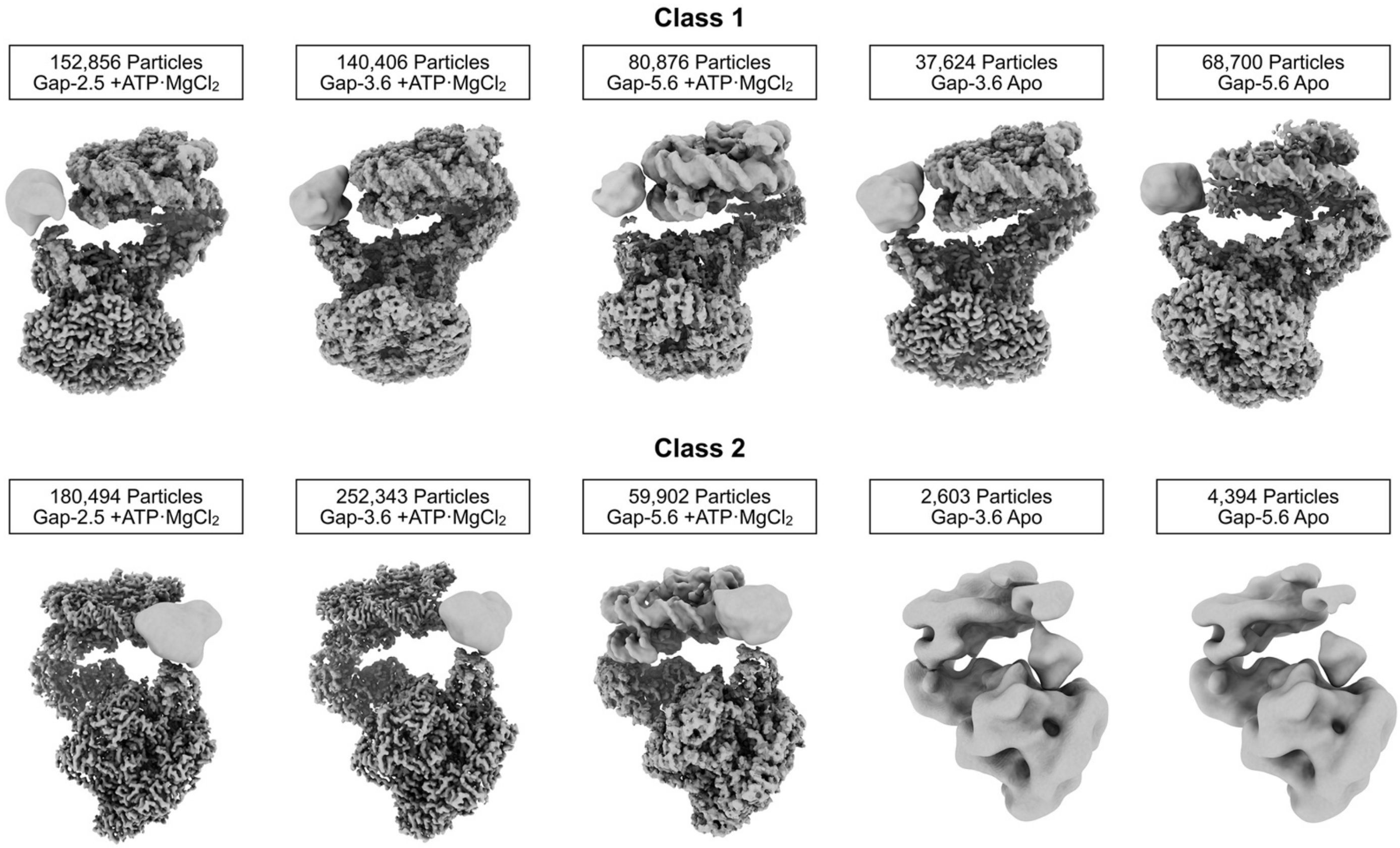

Supplementary Figure 4 Individual composite maps of Class 1 and 2 from each dataset.
Composite maps of Class 1 (upper row) and Class 2 (bottom row) of individual datasets, as marked. Particle numbers for each reconstruction are marked. All maps are displayed in the same orientation.

bioRxiv preprint doi: https://doi.org/10.64898/2026.08.11.744281; this version posted August 14, 2026. The copyright holder for this preprint (which was not certified by peer review) is the author/funder, who has granted bioRxiv a license to display the preprint in perpetuity. It is made available under a CC-BY 4.0 International license.

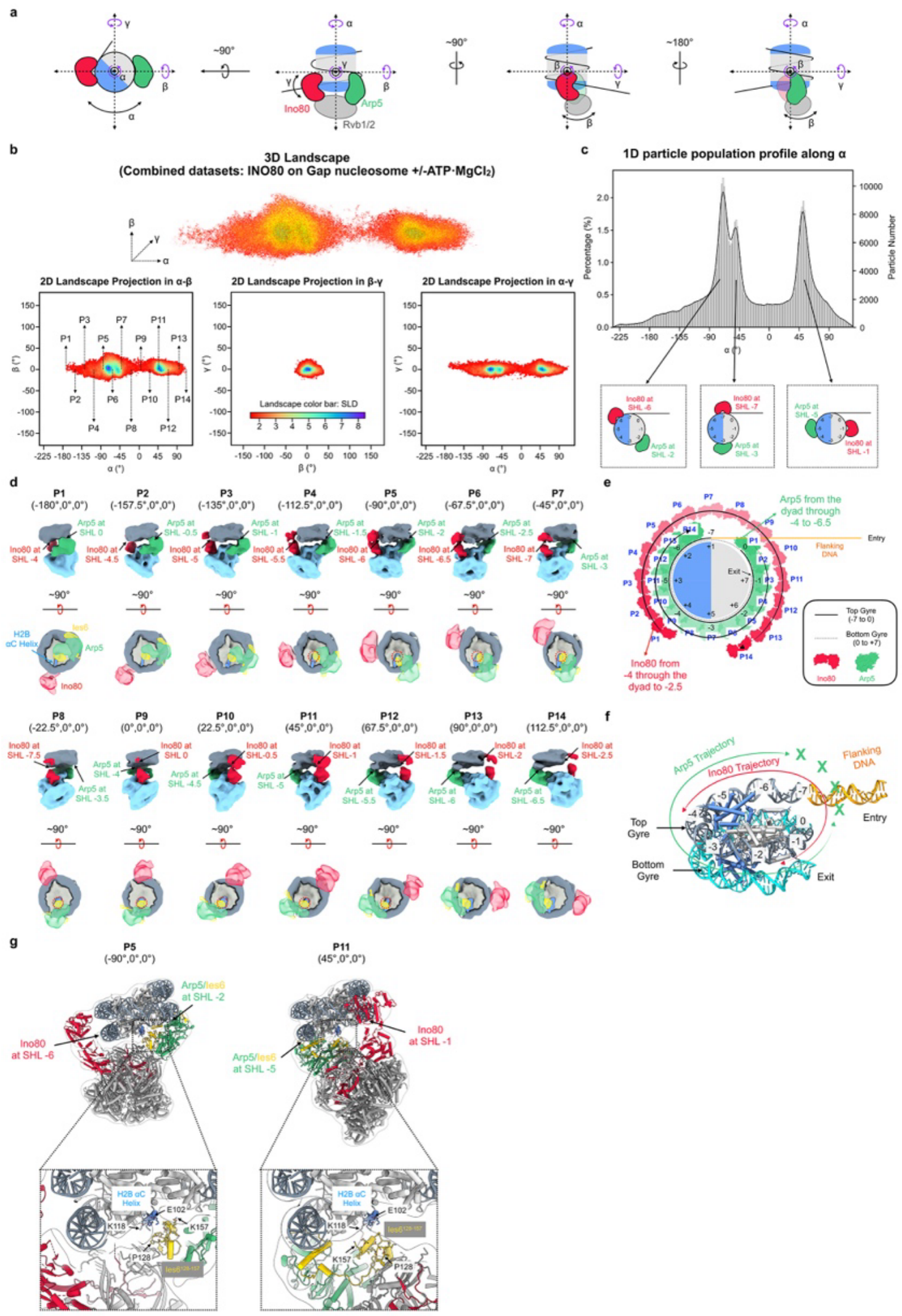

Supplementary Figure 5 The relative orientation landscape of all datasets combined, including Gap-2.5 ATP, Gap-3.6 ATP, Gap-5.6 ATP, Gap-3.6 Apo, Gap-5.6 Apo.

a) Schematic illustration of α, β, and γ angles in the canonicalized coordinate frame used to define the relative orientation between INO80 core and nucleosome. **b)** Upper panel: A 3D view of the landscape, with the direction of rotational axes marked. Each dot in the landscape represents a particle and its coordinate represent the relative orientation between INO80 core and nucleosome of that specific particle, and color coded with scaled local density (SLD). Particles with SLD less than 1 are filtered out. Bottom panel: 2D projection views of the 3D landscape onto the α-β, β-γ, and α-γ planes are shown. SLD = 1.5 was used to display projections. 14 positions with 22.5° intervals along a axis are marked in the α-β projection. **c)** Particle distribution profile along α axis derived from the landscape. Peaks are labeled as the location (SHL) of $Ino80^{ATPase}$ (red) and Arp5 (green) on nucleosome. **d)** Reconstructions calculated from particle extracted from the landscape at position marked as P1 to P14 along the α axis. The upper panels show side views, and the lower panels show top views from the direction of INO80 core, in which only densities of part of $Ino80^{ATPase}$ (red), Arp5 (sea green) and Ies6 (yellow) are shown. Densities of histone are in light grey DNA in dark grey. H2B αC helix of acidic patch is marked in cornflower blue. Red dotted circle marks the existence of a density connecting Ies6 and the H2B αC helix. **e)** Trajectories of $Ino80^{ATPase}$ (red) and Arp5 (green) illustrated on nucleosome with SHL position marked. The positions of $Ino80^{ATPase}$ and Arp5 are derived from reconstructions shown in (**d**). **f**) An illustration shows that the moving trajectory $Ino80^{ATPase}$ (red) and Arp5 (green) while keeping Arp5 on top gyre of nucleosomal DNA. **g)** Structural comparison of representative reconstructions corresponding to P5 (left, $Ino80^{ATPase}$ at SHL -6) and P11 (right, $Ino80^{ATPase}$ at SHL -1). Densities are displayed in low contour level as transparent. Insets highlight the interaction between the H2B αC helix and Ies2.

bioRxiv preprint doi: https://doi.org/10.64898/2026.08.11.744281; this version posted August 14, 2026. The copyright holder for this preprint (which was not certified by peer review) is the author/funder, who has granted bioRxiv a license to display the preprint in perpetuity. It is made available under a CC-BY 4.0 International license.

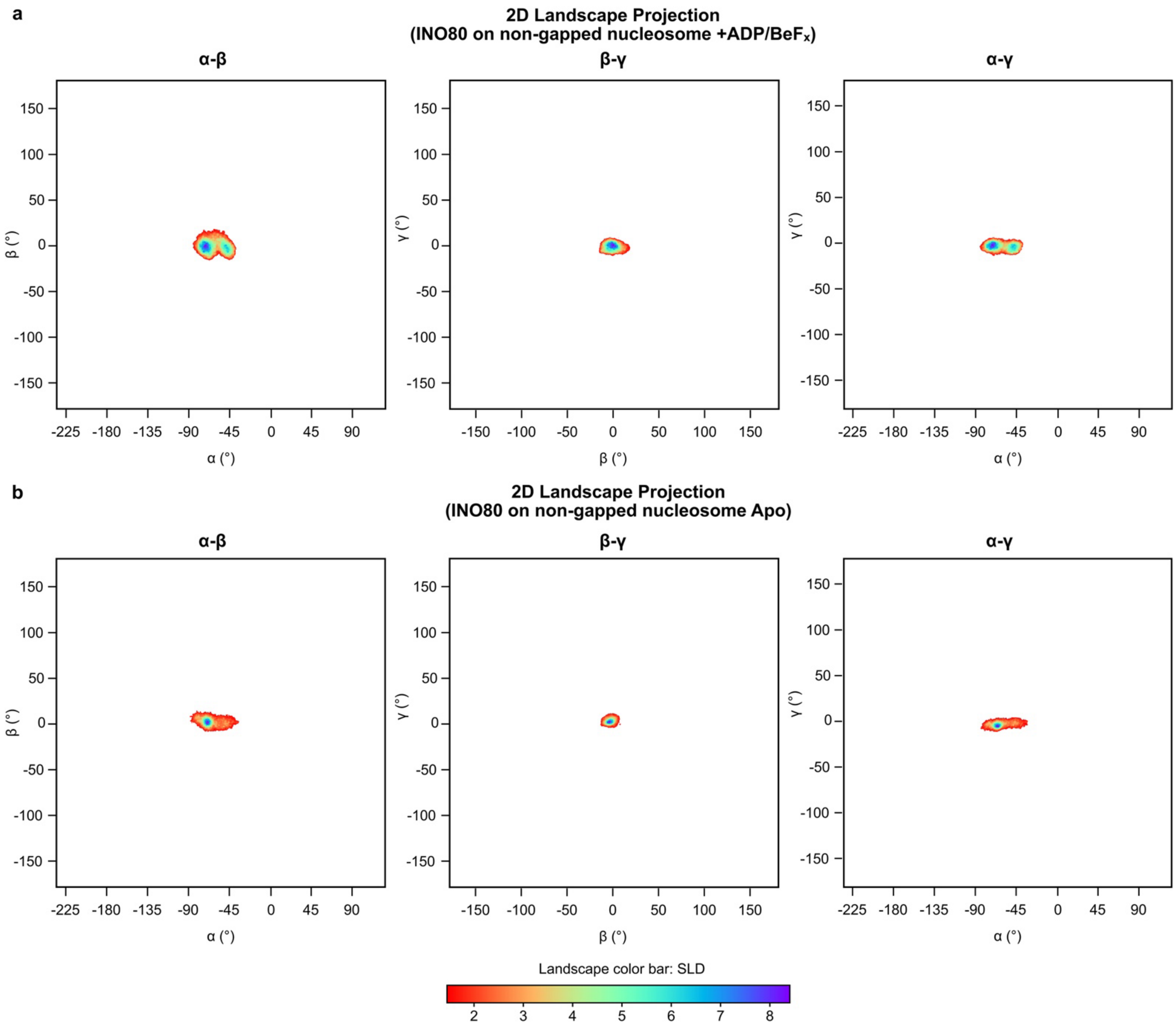

Supplementary Figure 6 Landscapes of the INO80/non-gapped nucleosome.
a) 2D projections of the landscape of INO80/non-gapped nucleosome in the presence of ADP/BeF_x onto the α-β, β-γ, and α-γ planes. **b)** 2D projections of the landscape of INO80/non-gapped nucleosome in the apo state onto the α-β, β-γ, and α-γ planes.

bioRxiv preprint doi: https://doi.org/10.64898/2026.08.11.744281; this version posted August 14, 2026. The copyright holder for this preprint (which was not certified by peer review) is the author/funder, who has granted bioRxiv a license to display the preprint in perpetuity. It is made available under a CC-BY 4.0 International license.

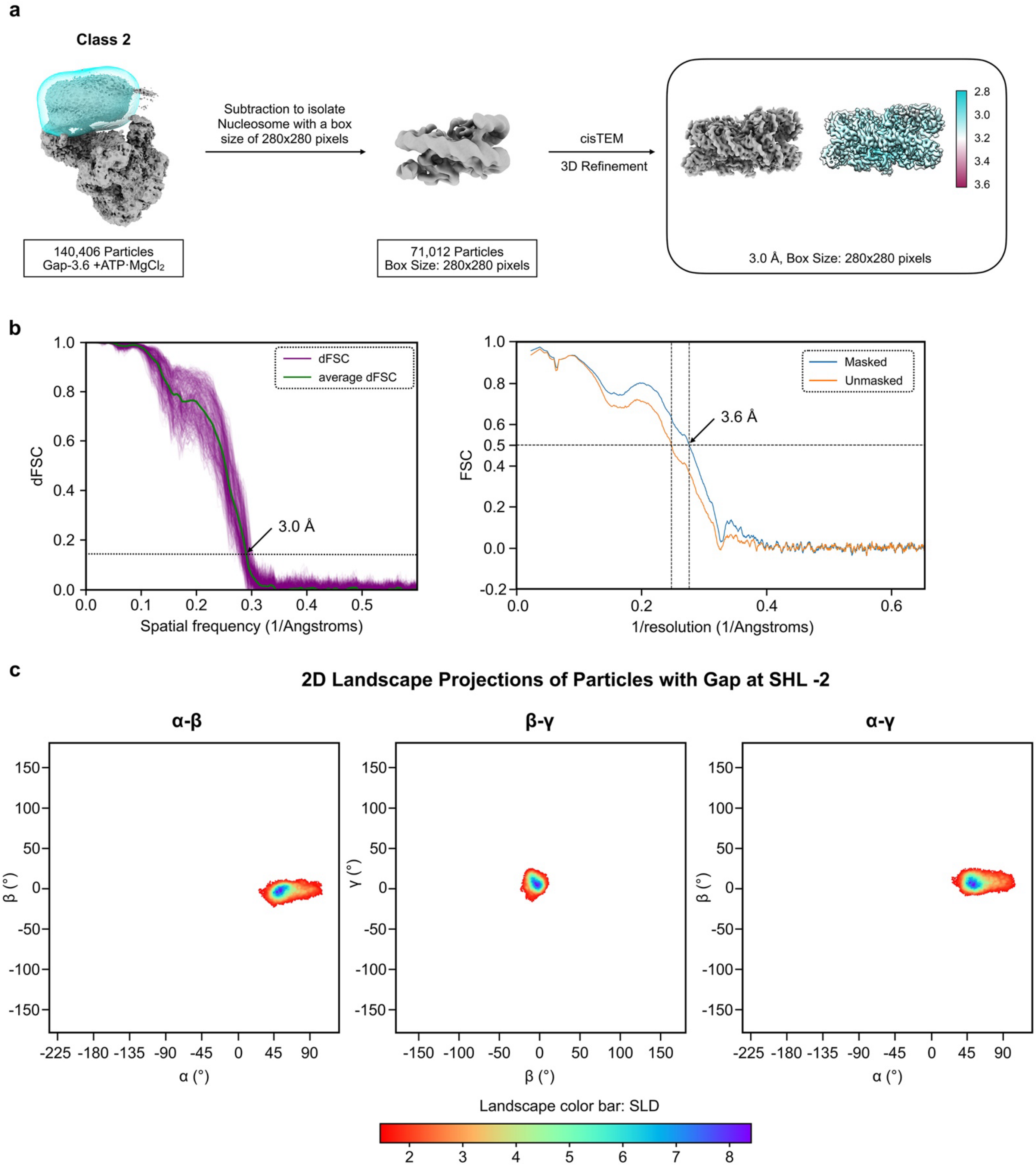

Supplementary Figure 7 Image Processing and landscape of particles with gap at SHL -2.
a) Image processing workflow of the Class 2 particle subset of the INO80/Gap-3.6 ATP dataset to identify particles with gap at SHL -2. **b)** Resolution estimation of the refined nucleosome map. dFSC and model to map FSC curves are shown. **c)** 2D projections in α-β, β-γ, and α-γ planes of the landscape of particles with gap at SHL -2.

bioRxiv preprint doi: https://doi.org/10.64898/2026.08.11.744281; this version posted August 14, 2026. The copyright holder for this preprint (which was not certified by peer review) is the author/funder, who has granted bioRxiv a license to display the preprint in perpetuity. It is made available under a CC-BY 4.0 International license.

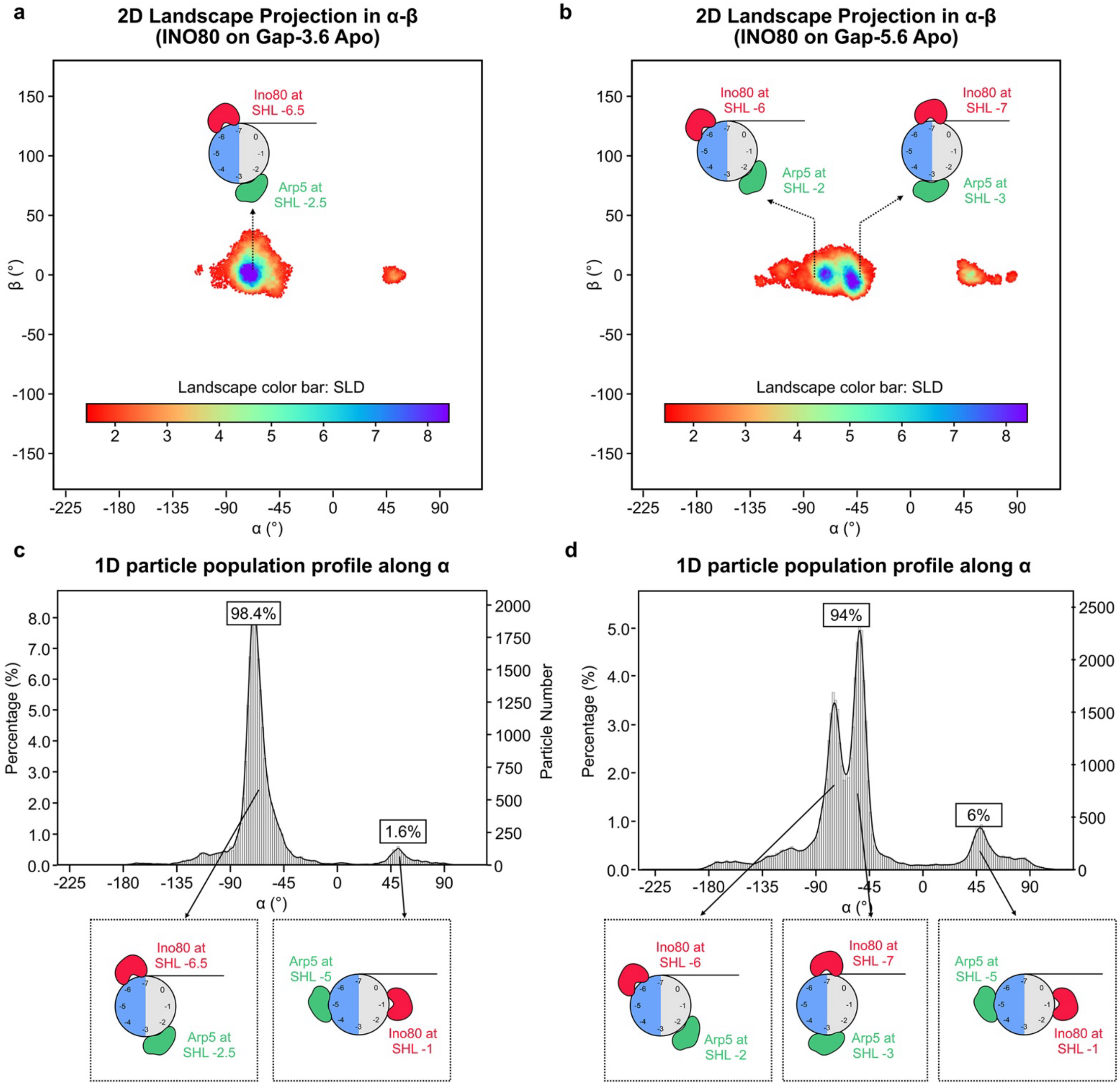

Supplementary Figure 8 Landscape of INO80/Gap-3.6 and INO80/Gap-5.6 in apo state.
a) and **b)** 2D projections of 3D landscapes onto the α-β plane for the INO80/Gap-3.6 apo (**a**) and INO80/Gap-5.6 apo (**b**). Orientations between INO80 core and nucleosome are illustrated as cartoon. Landscapes are colored by SLD as defined by the color bar. **c)** and **d)** Particle distribution profile along α derived from the landscapes shown in **(a)** and **(b)**. Percentage of particles under the peaks are marked. Insets show the ATPase and Arp5 binding positions corresponding to the peaks in the profile.

bioRxiv preprint doi: https://doi.org/10.64898/2026.08.11.744281; this version posted August 14, 2026. The copyright holder for this preprint (which was not certified by peer review) is the author/funder, who has granted bioRxiv a license to display the preprint in perpetuity. It is made available under a CC-BY 4.0 International license.

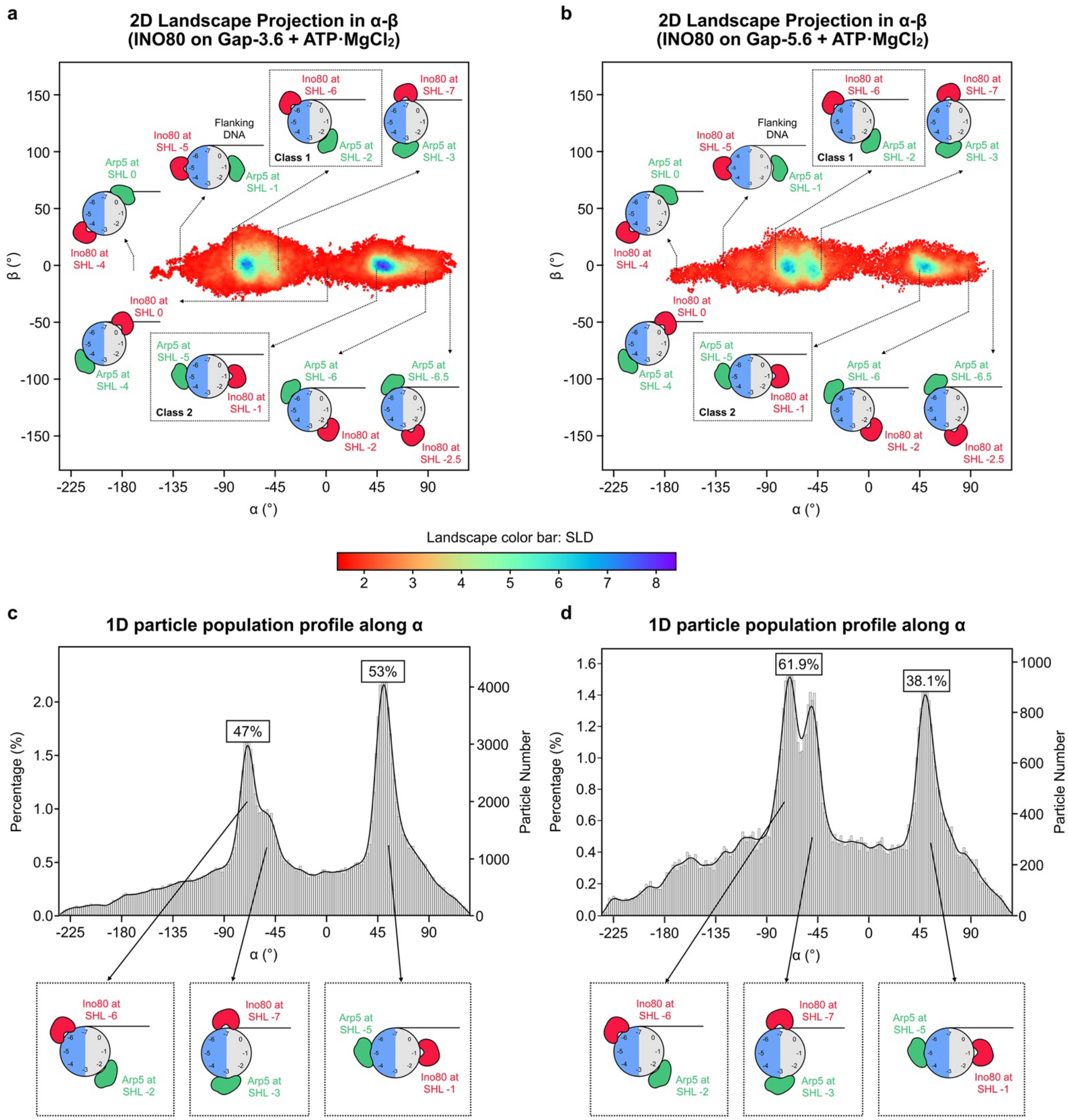

Supplementary Figure 9 Landscape of INO80/Gap-3.6 and INO80/Gap-5.6 in the presence of ATP. **a)** and **b)** 2D projections of 3D landscapes onto the α-β plane for the INO80/Gap-3.6 with ATP (**a**) and INO80/Gap-5.6 with ATP (**b**). Orientations between INO80 core and nucleosome from selected locations in the landscape are illustrated as cartoon. Landscapes are colored by SLD as defined by the color bar. **c)** and **d)** Particle distribution profile along α derived from the landscapes shown in **(a)** and **(b)**. Insets show the ATPase and Arp5 binding positions corresponding to the peaks in the profiles.

bioRxiv preprint doi: https://doi.org/10.64898/2026.08.11.744281; this version posted August 14, 2026. The copyright holder for this preprint (which was not certified by peer review) is the author/funder, who has granted bioRxiv a license to display the preprint in perpetuity. It is made available under a CC-BY 4.0 International license.

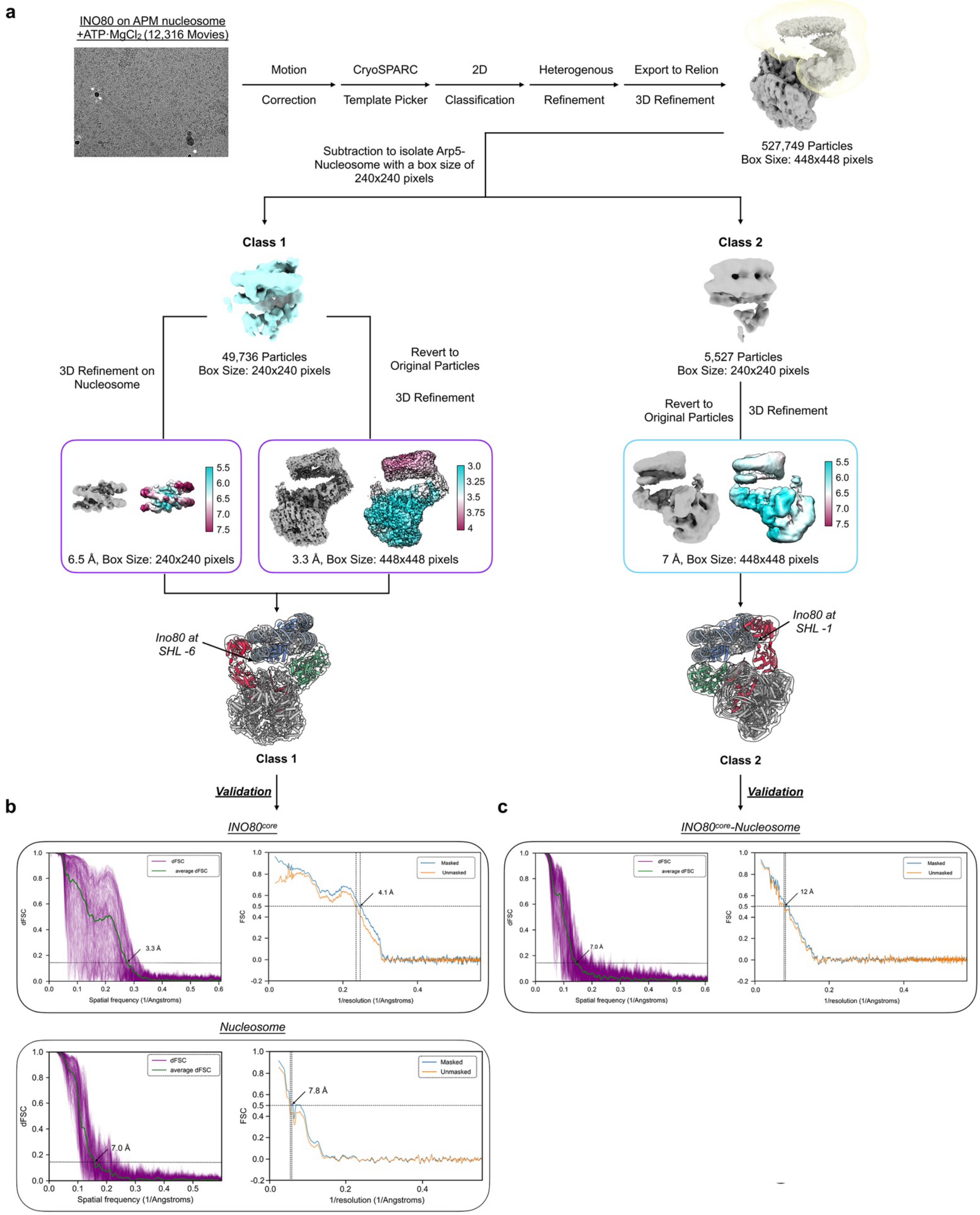

Supplementary Figure 10 Image processing flowchart of INO80/APM nucleosome with ATP.
a) Image processing flowchart of INO80/APM nucleosome with ATP. **b**) Resolution estimation of Class 1 INO80 and nucleosome from dFSC (left, 0.143 criterion) and model-to-map FSC (right, 0.5 criterion). **c**) dFSC (left, 0.143 criterion) and model-to-map FSC (right, 0.5 criterion) of Class 2 reconstruction.

bioRxiv preprint doi: https://doi.org/10.64898/2026.08.11.744281; this version posted August 14, 2026. The copyright holder for this preprint (which was not certified by peer review) is the author/funder, who has granted bioRxiv a license to display the preprint in perpetuity. It is made available under a CC-BY 4.0 International license.

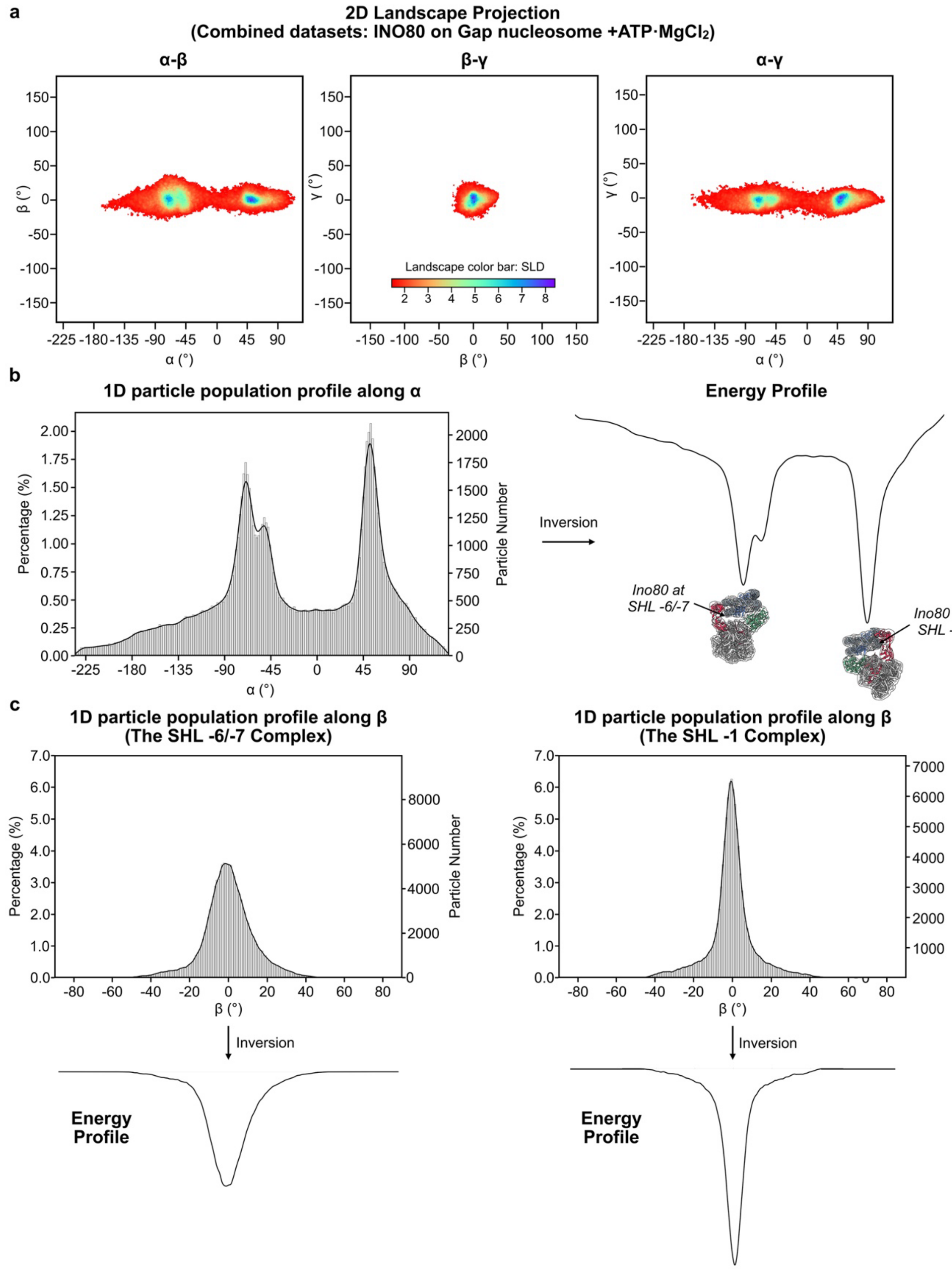

Supplementary Figure 11 Free energy landscapes predicted from the relative orientation landscape.
a) 2D projections of the relative orientation landscape of the combined INO80/Gap nucleosome ATP datasets onto the α-β, β-γ, and α-γ planes. **b)** Particle distribution profile along α (left) and a conformational free energy landscape generated assuming that the population distribution represents a near equilibrium distribution (right). With this assumption, particle distribution at various conformation is estimated to be inversely correlated with the free energy landscape and inversion of the particle distribution profile produces a corresponding conformational free energy landscape. **c)** Particle distribution profile along β axis for particles with $Ino80^{ATPase}$ at near SHL -6/-7 complex (left) and the SHL -1 complex (right). Together, they suggest that INO80 has a more stable interaction with nucleosome when $Ino80^{ATPase}$ is at near SHL -1 than that at SHL -6/-7.
